# Vermicomposting of food waste: assessing the stability and maturity

**DOI:** 10.1186/1735-2746-9-25

**Published:** 2012-12-13

**Authors:** Monireh Majlessi, Akbar Eslami, Hossein Najafi Saleh, Simin Mirshafieean, Sara Babaii

**Affiliations:** 1 Department of Environmental Health Engineering, School of Public health, ShahidBeheshti University of Medical Sciences, Tehran, Iran; 2 Department of Environmental Health Engineering, School of Public Health, Neyshabour University of Medical Science, Neyshabour, Iran; 3 Department of Environmental Health Engineering, School of Public health, Zanjan University of Medical Sciences, Zanjan, Iran

**Keywords:** Vermicomposting, Food waste, Stabilityindex, Maturityindex, Germination index

## Abstract

The vermicompost using earthworms (Eisenia Fetida) was produced from food waste and chemical parameters (EC, pH, carbon to nitrogen contents (C/N)) and germination bioassay was examined in order to assess the stability and maturity indicators during the vermicomposting process. The seed used in the germination bioassay was cress. The ranges of EC, pH, C/N and germination index were 7.5-4.9 mS/cm, 5.6-7.53, 30.13-14.32% and 12.8-58.4%, respectively. The germination index (GI) value revealed that vermicompost rendered as moderate phytotoxic to cress seed. Pearson correlation coefficient was used to evaluate the relationship between the parameters. High statistically significant correlation coefficient was calculated between the GI value and EC in the vermicompost at the 99% confidence level. The C/N value showed that the vermicompost was stable. As a result of these observations, stability test alone, was not able to ensure high vermicompost quality. Therefore, it appears that determining vermicompost quality requires a simultaneous use of maturity and stability tests.

## Introduction

The present trend about waste management is to focus on recycling and the recovery of waste as new materials or as energy. The waste organic materials produced by the city life, such as domestic refuse and food wastes are accumulating to become a significant amount. The vermicomposting of these by-products is more encouraged to avoid the loss of energy. Earthworms feed on the organics and convert material into casting rich in plant nutrients. The chemical analyses of casts show two times available magnesium, 15 times available nitrogen and seven times available potassium compared to the surrounding soil [1]. However, for safe application of vermicompost on soil, not only the quality of products should be including stable, but also the vermicompost must be mature.

Problems associated with compost or vermicompost phytotoxicity– the “intoxication of living plants by substances present or produced in the growth medium, when these substances are taken up and accumulated in plant tissue” [2], have impeded compost use in agriculture [3-6]. The source of materials and the biological activity associated with composting and vermicomposting make it extremely difficult to assess the rate of compost/vermicompost application and its suitability as a soil amendment.

Maturity is a term used to indicate the level of phytotoxic substances in composts and compost suitability for plant growth [7-9]. The land-applied immature compost or vermicompost gives rise to a serious N-deficiency in crops, and the rapid decomposition of immature compost causes a decrease of the oxygen concentration around the root system [10,11]. Additionally, land-applied immature compost inhibits the plant growth by the production of phytotoxic substances, fundamentally ammonia, ethylene oxide, and organic acids [12].

Some of the parameters that have been used to measure the maturity include changes in nitrogen species, pH, optical density, temperature, specific gravity, plant assays, respiration, microbial population changes [13], specific oxygen uptake rate(SOUR) Scaglia et al. [14], Solvita test [15] and activity of cellulolytic microorganisms [16]. Two additional methods often used for measuring compost maturity include C/N ratio and electrical conductivity (EC). Composts with high C/N ratio can cause nitrogen immobilization upon amendment to soil [17] and those with low C/N ratio can cause ammonium toxicity [13,17]. Researchers have suggested various ideal C/N ratios ranging from <12 to <25 [4,9,18], but the optimal value is often dependent on the initial feedstock [8,13]. Electrical conductivity measures the concentration of soluble ions or the salinity of the compost. Excessive salinity in compost can directly cause phytotoxicity, depending on the salt tolerance of the plant species. Salinity can be also developed from nitrogen mineralization and production of organic acids. However,these factors indicate the compost stability and not its maturity. The simple method to evaluate maturity, phytotoxicity, is germination test and plant bioassay.

The bioassays of phytotoxicity has received great attention by environmental agencies of world. The phytotoxicity effects produced by organic wastes are the result of a combination of several factors, like the presence of heavy metals, ammonia, salts and low molecular weight organic acids [19]. The evaluation of organic waste toxicity by biological testing is therefore extremely important for screening the suitability of wastes for land application.Therefore, use of plant seeds to indicate compost or vermicompost maturity is seen as a protective approach, since respiration or stability testing does not directly indicate potential plant problems. However, maturity is in part, affected by the relative stability of the material. For example, the poorly stabilized composts are adequately correlated with seed germination indices; however, this is not true with highly stable composts.

The objects of this study are determination of stability and maturity indices of vermicompost and analysis of these indices for determining of appropriate time of vermicompost application on soil. Phytotoxicityis represented as cress seed germination and stability indicators include electrical conductivity, C/N ratio and pH in vermicompost. Results from food waste vermicompost are presented.

## Materials and methods

### Earthworm culture

Composting earthworms, i.e., *Eisenia fetida* of different age groups were obtained from ZESCO, Zanjan, Iran,where it has been cultured for the last 5 years. Stock earthworms were cultured, in the laboratory, on partially decomposed cow dung for 4 months.

### Collection of food waste

The food waste used as substrate was collected from Shaheed Beheshti University of Medical Sciences’ restaurant, on which the source separation can be done easily. The food waste was collected for a week and precomposted for 18 days prior to vermicomposting for thermal stabilization and initiation of microbial degradation. In precomposting stage, the pile of waste was aerated with the continuous aeration by the fan connected to a polyethylene pipe with pore diameter of 0.5 cm on the body for airflow distribution in the pile. The total volume of collected food waste at the end of the week was 236.8 liters.

### Experimental setup

The experiments were conducted in plastic pots (three pots), each with capacity of 2.5 kg waste, with a small hole at the bottom. 500 g of waste was taken in each pot along with 300 g of vermicompost in bottom and top of pots to provide an initial favorable environmental condition for the worms. Fifty healthy earthworms of the same size (*E*. *fetida*) were introduced in each of plastic pots. The moisture content was maintained between 65% and 75% during the study by periodically sprinkling of an adequate quantity of water. The duration of experiments was seven weeks.

### Physicochemical analysis

The chemical analysis of raw organic waste used and vermicompost samples, collected weekly, was done for total organic carbon (TOC), total Kjeldahl nitrogen (TKN), electrical conductivity (EC) and pH. Vermicompost samples were digested for TKN. TKN was analyzed by micro-Kjeldahl titrimetric method on 0.1 g samples [20]. Total volatile solids (TVS) were determined as sample weight loss (previously oven-dried at 105°C) upon ashing at 550°C for 2 h in a muffle furnace. Total organic carbon was calculated by multiplying the TVS values by 1.8 [21,22].Water extracts of the vermicompost samples were prepared by shaking the fresh sample with distilled water at 1:10 w/v (dry weight basis) for 30 min at room temperature by filtration. The fresh extracts were subjected to pH and EC measurements.

### Germination bioassay (Phytotoxicity assay)

The germination bioassays followed a modified procedure, based on Zucconi and de Bertoldi [23]. Approximately 15–25 g (ww) of vermicompost was mixed with deionized water in an Erlenmeyer flask at a mixing ratio of 10:1 (water volume, in mL, to dry weight, in g); Mixing was performed for 15 min at room temperature. The slurry was then filtered under vacuum using aWhatman No. 6 filter paper. 10 cress seeds (*Lepidium sativum*) were placed on filter paper Whatman No.1 in a 100mm diameter Petri dish during each run. The above number of seeds resulted in enough space to facilitate seed growth and the measurements that followed. Then, 5mL of vermicompost filtrate was added on each Petri dish. The test was run in triplicate. A different vermicompost filtrate was prepared every time during each of the three replicate runs. Controls,also, run in three replicates for cress seed, were prepared with deionized (DI) water. Petri dishes were incubated at 25°C. The aforementioned temperature was within the optimum temperature germination range for cress seeds used throughout the study. Germination percentage and root length were measured after an incubation period of two days and were expressed as a percentage of the corresponding values of the control Zucconi and de Bertoldi [23]. Seeds with root lengths less than 2 mm were not rendered to germination. According to the above description, GI was calculated based on the following formula:

(1) $$\begin{array}{c} \mathrm{GI}\left(\%\right)=\frac{\mathrm{Seed}\;\mathrm{germination}\times \mathrm{root}\;\mathrm{length}\;\mathrm{of}\;\mathrm{treatment}\;}{\mathrm{Seed}\;\mathrm{germination}\times \mathrm{root}\;\mathrm{length}\;\mathrm{of}\;\mathrm{control}}\\ \;\times 100 \end{array}$$

### Statistical analysis

The statistical analysis of data was carried out using the SPSS 16.0 program for Windows. Pearson correlation coefficient was used to evaluate the relationship between the parameters.

## Results

### Physicochemical properties

Table 1 summarizes data pertaining to TOC, TKN, and C/N ratio during vermicomposting process. The initial TOC, TKN and C/N ratio of food waste prior to vermicomposting were 46.70%, 1.55% and 30.13. Data revealed a decrease of C/N ratio from 30.13 to 14.32 at the end of process. Also, total organic carbon and nitrogen contents decreased as a result of vermicomposting process; but the decrease rate of nitrogen content was slow.

**Table 1:** Chemical analysis of vermicompost produced with food waste

**Week**	**TKN**	**TOC**	**C:N**
0	1.55	46.70	30.13
1	1.45	41.90	28.9
2	1.28	34.81	27.2
3	1.12	27.88	24.9
4	1.2	25.51	21.26
5	1.4	24.83	17.74
6	1.21	19.91	15.40
7	1.1	15.75	14.32

*All values are given in percentage except C/N ratio.

**Figure 1:**
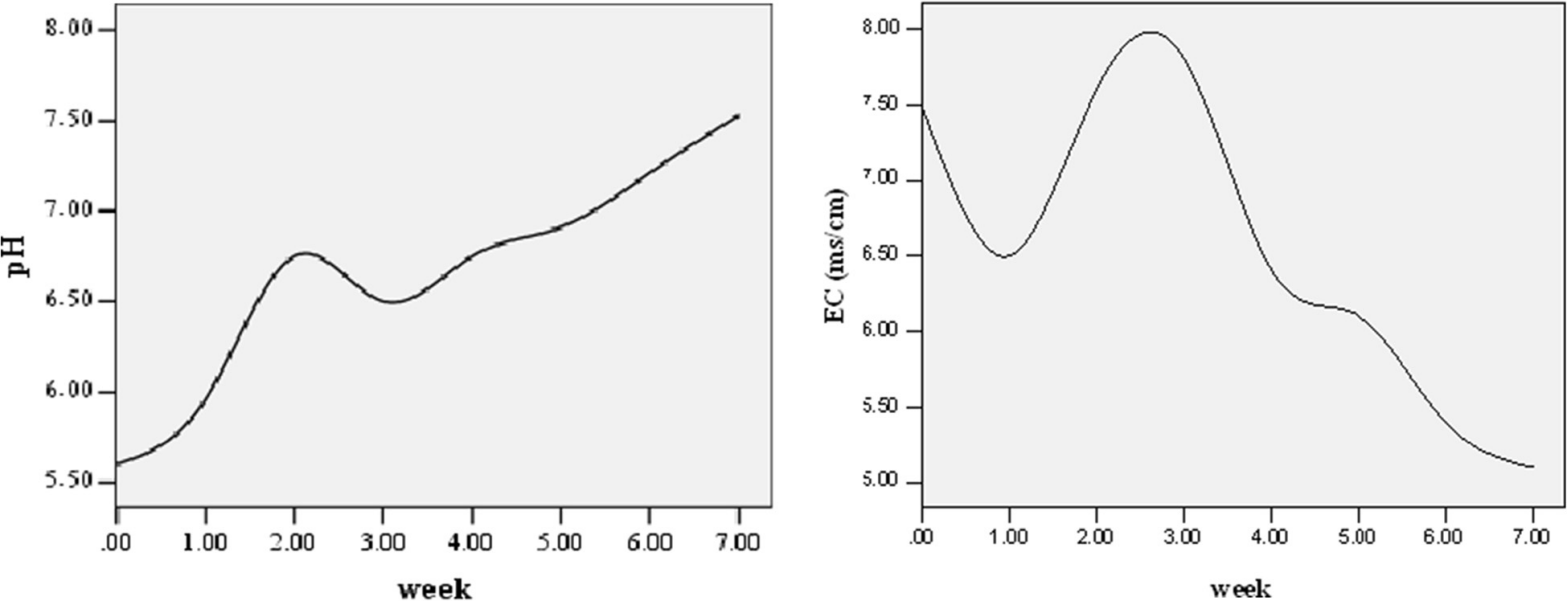
Changes in pH and EC values monitored during the vermicomposting process.

Figure 1 shows the changes of EC and pH in selected times. The pH of food waste increased during the vermicomposting process and changed from 5.6 in the first week to 7.53 at the end of 7th week. The initial EC value was 7.5 mS/cm and decreased to 4.9 mS/cm at the end of the vermicomposting process.

### Germination bioassay (Phytotoxicity bioassay)

Table 2 shows the result of the germination test with cress seeds (*Lepidium sativum*). The germination index was 12.8% in the food waste prior to vermicomposting process, and reached a value of 58.4% at the end of the process. A decrease in germination index value occurred between the first to 3th weeks.

**Table 2:** Changes in the germination index of the vermicomposting material collected at different times

**Week**	**0**	**1**	**2**	**3**	**4**	**5**	**6**	**7**
GI(%)	12.8±0.67	19.7±0.55	17.4±0.53	13.5±0.35	23.3±0.66	36.2±0.43	41.6±0.29	58.4±0.28

Values are means ± standard error (n = 3).

### Linear correlations

EC showed a high and significant inverse correlation with GI (Table 3). A strong inverse correlationwas found for C/N with GI (Table 3). Significant and negative correlations were found for TOC with GI.

**Table 3:** **Pearson correlation coef**fi**cients among various parameters**

	**Maturity index**	**Stability indices**				
	**GI Crs**	**EC**	**N**	**C**	**pH**	**C**/**N**
GI Crs.	1	−0.924**	n. s.	−0.800*	0.827*	−0.904**

n.s.: not significant; Crs.: cress; C: total carbon content (% dry weight); N: total nitrogen content (% dry weight).

*Significant at p < 0.05.

**Significant at p < 0.01.

## Discussion

### Physicochemical changes

During vermicomposting of food waste, as shown in Table 1, a significant decrease revealed in percentage of the TOC from 46.70 to 15.75; whereas the total N content changed slightly with the vermicomposting time. This may be due to existance of labile organic compounds, such as simple carbohydrates, fats and amino acids in the food waste that are degraded quickly in the first stage of vermicomposting. In addition, obtained results from Table 1 showed that organic substrates degraded at a lower rate and TOC content decreased slightly at the end of the process. This can be due to existing of resistant organic substrates, such as cellulose, hemicellulose and lignin in the process that are degraded partially. Bernal et al. [24], mentioned that vermicomposting or composting involves a partial mineralization of the organic substrate, leading to carbon losses throughout the process.

The results of Table 1 showed that C/N ratio decreased from 30 to almost 14. A significant reduction of this ratio during the vermicomposting process was mainly due to the depletion of easily degradable carbon compounds and C losses as CO_2_. Also, results from vermicomposting process showed that process duration (7 weeks) was sufficient for stability of vermicompost because the C/N ratio decreased to about 14. Our data are supported by [12]. According to Brandon findings, the stabilized compost has a C/N ratio from 10 to 15.

A general increase in pH value recorded during the vermicomposting process, suggests the alkalinization of the food waste because of the release of ammonia from the degradation and mineralization of organic compounds (Figure 1). However, the results showed a drop in pH between the second and third weeks.This may be due to development of anaerobic conditions and accumulation of organic acids in parts of pot. Our results are supported by (Rostami et al., 2010). Acceptable pH for the microorganisms is generally in the range of 6.0–7.5 Boulter-Bitzer et al. [25]. The pH value of ourend product was found in the optimum range. Neutral and partial alkaline pH values are usually indicators of stable vermicomposts, since pH is known to increase in the latter stages of vermicomposting.

In the beginning of the vermicomposting, electrical conductivity value of food waste was 7.5 mS/cm. This value reached to 4.9 mS/cm in the final product (Figure 1). According to the obtained results, it was observed that this parameter increased between the second to third weeks probably due to the release of soluble salts like ammonium and phosphate, resulting from the decomposition of easily biodegradable organic substrates. Similar results were shown by [26] in composting of hog manure. He found that salinity could develop from nitrogen mineralization and production of organic acids. The EC affects the quality of compost and vermicompost in a large way because it reflects their salinity and suitability for crop growth. Mengel *et al*. [27] observed that excessive salinity in compost could cause phytotoxicity directly, depending on the salt tolerance of the plant species.

### Evaluation of maturity

The initial germination index (GI) of food waste was 12.8±0.67. The GI of vermicompost increased during the vermicomposting process (Table 2). This can be due to well progress of decomposition of organic substrates and reduction of phytotoxic compounds resulting from vermicompost ageing. However, in the present study, germination of cress seeds in the vermicomposting material extracts was 58.4% after 48 h at the end of process, which presented moderate phytotoxicity resulting from high electrical conductivity value in the vermicompost and sensitivity of cress to salinity. Our data are supported by Paradelo *et al*. [28] which mentioned that GI values between 50% and 80% mean moderate phytotoxicity.

### Relation between maturity and stability indices

A potential relation between the maturity index (GI) and the stability indices (C/N, EC and pH) of vermicompost were sought. Table 3 includes the Pearson correlation coefficients (r) among the parameters, namely GI, C/N ratio, C content, N content, pH and EC. Some correlation coefficients are negative and some are positive. The negative signs indicate that as the carbon content or C/N ratio increases, germination index decreases, that is phytotoxicity increases. In addition, EC was found to have a negative statistically significant correlation with the germination index of cress (r = −0.924); this is a likely indication of the sensitivity of this seed to vermicompost with high amounts of salts. Salinity can have a detrimental effect on seed germination and plant growth, especially in the seedling stage. In general, salinity effects are mostly negligible in extracts with EC readings of 2mS/cm or less [29,30]. This critical level was exceeded in all the food waste extracts in duration of vermicomposting process.

The C/N ratio had an expected negative statistically significant correlation with GI. No significant correlation was achieved between the N content and the GI from cress, which might be an indication of the non-sensitivity of cress seed to vermicompost with these values of nitrogen (Table 3). Statistically significant correlation was found between the GI from cress and the carbon content, which indicates the likely sensitivity of the cress seed to wastes with potentially high biodegradability.Statistically significant positive correlation was found between the GI from cress and the pH value, which indicates the likely stability of vermicompost. Wu et al. [22] reported that pH and EC changes were caused by decomposition of organic acids, suggesting that simple parameters such as pH and EC might be good indicators of compost stability.

## Conclusion

Finally, it should be stated that a stability test alone, is not adequate to ensure high vermicompost or compost quality. For example, vermicompost was moderately phytotoxic to cress seeds, and, therefore, rather immature, but resulted in a low C/N ratio, which is indicative of stable vermicompost. Therefore, it appears that determining vermicompost quality requires a simultaneous use of maturity and stability tests. In addition, maturity in vermicompost is not the same as in compost. For example, one of the new methods for maturity evaluation is Solvita test. The Solvita test is actually a way to measure CO_2_ released from microbial activity. However, in the vermicomposting process, this can serve to indicate the worms’ activity. Therefore, it appears that use of germination test is suitable for evaluating of vermicompost maturity as a simple and inexpensive test.

## Competing interests

The author’s have no competing interests’ for this article.

## Authors’ contributions

HNS conceived of the study, and participated in its design and draft the manuscript and performed the statistical analysis. MM participated in the design of study. AE coordinated and help to draft the manuscript. SM coordination and helped to physicochemical analysis. SB coordination and helped to earthworm culture and helped to carry out the germination bioassay study. All authors read and approved the final manuscript.
